# Clinical workplace learning: perceived learning value of individual and group feedback in a collectivistic culture

**DOI:** 10.1186/s12909-018-1188-0

**Published:** 2018-04-19

**Authors:** Yoyo Suhoyo, Johanna Schönrock-Adema, Ova Emilia, Jan B. M. Kuks, Janke Cohen-Schotanus

**Affiliations:** 1 Department of Medical Education, Faculty of Medicine, Public Health, and Nursing, Universitas Gadjah Mada, Gd. Prof. Drs. Med. R. Radiopoetro, Lt. 6 Sayap Barat, Jl. Farmako, Sekip Utara, Yogyakarta, 55281 Indonesia; 2 Institute for Medical Education, University of Groningen and University Medical Center Groningen, Groningen, The Netherlands; 3 Department of Obstetrics and Gynecology, Faculty of Medicine, Public Health, and Nursing, Universitas Gadjah Mada, Yogyakarta, Indonesia; 4 Department of Neurology, University of Groningen and University Medical Center Groningen, Groningen, The Netherlands

**Keywords:** Clinical workplace learning, Group feedback, Individual feedback, Collectivistic culture

## Abstract

**Background:**

Feedback is essential for workplace learning. Most papers in this field concern individual feedback. In collectivistic cultures, however, *group* feedback is common educational practice. This study was conducted to investigate the perceived learning value and characteristics of individual and group feedback in a collectivistic culture.

**Methods:**

During two weeks, on a daily basis, clerkship students (*n* = 215) from 12 clinical departments at Faculty of Medicine, Universitas Gadjah Mada, Yogyakarta, Indonesia, recorded individual and group feedback moments by using a structured form: the providers, focus and perceived learning value of feedback. Data were analysed with logistic regression and multilevel techniques.

**Results:**

Students reported 2687 group and 1535 individual feedback moments. Group feedback more often focused on history taking, clinical judgment, patient management, patient counselling, and professional behaviour (OR ranging from 1.232, *p* < .01, to 2.152, *p* < .001), but less often on physical examination (OR = .836, *p* < .01). Group feedback less often aimed at correcting performance deficiencies (OR = .523, *p* < .001) and more often at comparing performance to the standard (OR = 2.447, *p* < .001) and planning action to improve performance (OR = 1.759, *p* < .001). Group feedback was perceived as more valuable than individual feedback (M = 4.08 and 3.96, respectively, *β*_*group*_ = .065, SE = .026, *p* < .01).

**Conclusion:**

In collectivistic cultures, group feedback may add to the array of educational measures that optimize student learning. Congruence between culture and type of feedback may be important for the effectiveness of feedback.

## Background

Students need feedback during clerkships to improve their learning and clinical competencies [1–6]. Most studies about the effectiveness of feedback in the clinical work place focus on *individual* feedback to students. However, there are cultures in which *group* feedback is a common educational practice [7]. Group feedback is defined as ‘feedback from one teacher to more than one student at the same moment, with a message that is based on the individual performance of anonymous students’. The question arises whether group feedback adds to clinical workplace learning as individual feedback does.

Based on a literature review of feedback studies, feedback in the clinical setting was defined as *‘specific information about the comparison between a trainee’s observed performance and a standard given with the intent to improve the trainee’s performance’* [8]. Research has shown that feedback in a clinical setting is more effective if the following requirements are met: the feedback is specific in content by focusing on observable competencies, compares student performance to a standard, corrects performance deficiencies, and contains a plan of action to improve performance [8]. However, these requirements are mainly derived from research with a focus on *individual* feedback. Whether these requirements also apply to *group* feedback has – to the best of our knowledge – not yet been investigated.

Individualism has been acknowledged as a cultural dimension influencing feedback processes [7, 9, 10]. In collectivistic cultures, teachers usually deal with students as a group rather than as individuals, also when they give feedback [7, 11]. The question arises what the perceived learning value is of individual versus group feedback in collectivistic cultures. In this study, we therefore investigated in a collectivistic culture how students perceive the learning value of individual and group feedback during clerkships. Besides, we compared individual and group feedback on the focus of feedback and on how often the requirements for effective feedback (as defined for individual feedback) were satisfied. Our research questions were:

- How often do students receive individual or group feedback during clerkships?
- Are there any differences between individual and group feedback with regard to the focus of feedback and regarding how often the requirements for effective feedback (as defined for individual feedback) are satisfied?

Some studies showed that students value feedback from specialists differently than feedback from residents [10, 12–14]. For instance, Indonesian students perceived feedback from specialists as more instructive than feedback from residents [10]. Therefore, we also took the feedback provider into account in our study.

## Methods

### Context

This study was conducted at Faculty of Medicine, Universitas Gadjah Mada, Yogyakarta, Indonesia. Indonesia is classified as low on individualism and is, therefore, considered as a collectivistic country. [11] Data have been obtained in the final part of the five-year medical curriculum, when students had to rotate through 12 clerkships at the main teaching hospital and/or one or more of the eleven affiliated hospitals. Several teaching-learning and assessment methods that facilitate individual or group feedback were used such as bed side teaching, morning report, case based discussion, clinical skill assessment using logbook, and professional behaviour assessment.

### Participants and procedure

We asked students (*n* = 286) from 12 clinical departments (surgery, internal medicine, paediatric, obstetrics and gynaecology, neurology, ophthalmology, psychiatric, dermatology, otorhinolaryngology, radiology, medical forensic, and anaesthesiology) to record – for 2 weeks on a daily basis – all moments on which they received feedback on their performance. For each feedback moment, students had to note down:

- whether the feedback was given to them as an individual or as a group of students;
- the kind of feedback provider: specialist or resident;
- the clinical competencies on which the feedback was focused: history taking, physical examination, clinical judgement, patient management, patient counselling and/or professional behaviour;
- whether the feedback provider (1) compared student performance with a standard such as a protocol, guideline, standard of medical services, standard operating procedure or clinical skills’ book that used or recommended in each clinical department, (2) gave information to correct performance deficiencies (i.e. mentioned the student’s weaknesses and then explained what the correct performance looks like/implies), and (3) planned action to improve performance;
- the perceived learning value of the feedback on a 5-point Likert scale (1 = not valuable to 5 = very valuable).

To record feedback, we used a structured form that has been piloted before (*n* = 19) to check its applicability. A letter explaining the purpose and the procedure of the research, and the definition and example of feedback and each focus and characteristic of feedback accompanied the form. Students completed the structured form anonymously. We obtained ethical approval for this study from the Medical and Health Research Ethics Committee (MHREC) at Universitas Gadjah Mada.

### Data analysis

We conducted logistic regression to examine differences between individual and group feedback and between specialists and residents with regard to the focus and characteristics of feedback. In addition, we investigated whether there were any interactions between feedback type and feedback provider. We used multilevel analysis to examine whether the perceived learning value of individual and group feedback differed (MLwiN version 2.01). In multilevel analysis, we analysed feedback moments as measurements that were nested within students because each student reported several and a varying number of feedback moments. We conducted a multilevel analysis in two stages: (1) we estimated the empty model, which describes the variation in learning value associated with feedback moments and students separately; and (2) we used the main effects model to calculate the impact of feedback type – individual or group feedback – on perceived learning value. Then, the difference in deviance between the models was used to assess the fit of the main effects model to the data. We analysed differences using a chi-square test with the degrees of freedom being equal to the number of parameters added.

## Results

The results are summarized in Table 1. Of these participants, 71 were excluded for having completed their forms inaccurately or incompletely. In total, 215 students (75%) reported 4222 feedback moments during the 2 weeks. Of these, 2687 moments were reported as group feedback (64%, average 6.3 per student per week) and 1535 as individual feedback (36%, average 3.6 per student per week). Group feedback was more often given by specialists (66%) and individual feedback more often by residents (66%).

**Table 1:** The percentages of individual and group feedback moments, provided by specialists or residents, that include the focus of feedback specified in the first column and/or that satisfy the specific characteristics of feedback, and differences between these percentages^a^

Characteristic feedback	The percentage of reported feedback moments (Number of Students = 215)				Logistic regression			
	Individual feedback		Group feedback					
	specialist (nfm = 515) (%)	resident (nfm = 1020) (%)	specialist (nfm = 1760) (%)	resident (nfm = 927) (%)	predictor^b^	*e*^β^ (OR)	95% CI	
							Lower	Upper
Focus feedback								
History taking	40	39	46	44	ft	1.257*	1.100	1.438
Physical examination	61	61	56	57	ft	.836*	.731	.956
Clinical judgment	61	51	63	58	ft	1.232*	1.078	1.407
					fp	.764**	.671	.869
Patient management	46	42	54	51	ft	1.435**	1.257	1.639
Patient counselling	14	14	22	14	ft	1.708**	1.303	2.238
					fp	.996	.736	1.347
					ft*fp	.587*	.405	.851
Professional behaviour	16	17	30	17	ft	2.152**	1.667	2.777
					fp	1.019	.765	1.357
					ft*fp	.470**	.331	.666
Compare students’ performance to the standard	30	33	52	42	ft	2.447**	1.984	3.017
					fp	1.125	.895	1.414
					ft*fp	.602**	.455	.795
Information on correct performance deficiency provided	72	70	55	57	ft	.523**	.456	.602
Plan to improve performance provided	17	16	28	24	ft	1.759**	1.489	2.078

*p < .01; **p < .001

^a^*OR* Odds Ratio, *CI* Confidence Interval, *ft.* feedback type (0 = individual, 1 = group), *fp* feedback provider (0 = specialist, 1 = resident), ft.*fp interaction feedback type*feedback provider, *nfm* number of feedback moments

^b^only significant predictors were included in the table

### Focus of feedback

Compared to individual feedback, group feedback was more often reported as focusing on history taking, clinical judgment, patient management, patient counselling, and professional behaviour (OR ranging from OR = 1.232, *p* < .01 to OR = 2.152, *p* < .001), whereas individual feedback was focused more on physical examination (OR = .836, *p* < .01). Closer inspection of the outcomes shows that two of these findings were attributable to significant interactions between feedback type and feedback provider: in group feedback, specialists were reported to focus more often on patient counselling (OR = .587, p < .01) and professional behaviour (OR = .470, *p* < .001) than residents were. We did not find such differences in case of individual feedback. Furthermore, specialists were reported to focus their feedback more often on clinical judgement than residents were, both in group and in individual feedback (OR = .764, *p* < .001).

### Characteristics of feedback

Students reported that, in group feedback, their behaviour was significantly more often compared with the standard than it was in individual feedback (around 50%, OR = 2.447, *p* < .001), and this held especially if the feedback was provided by specialists (OR = .602, *p* < 0.01). Information on performance deficiencies was more often provided in the individual feedback situation (OR = .523, *p* < .001), whereas actions to improve student performance were more often planned in group feedback (OR = 1.759, *p* < .001).

### Perceived learning value of feedback

Overall, respondents perceived group feedback as more valuable than individual feedback (M = 4.08 and 3.96, respectively, *β*_*group*_ = .065, SE = .026, *p* < .01, not shown in the table).

## Discussion

Our study in Indonesia, a country classified as a collectivistic culture [11], showed that during clerkships, group feedback was more often provided than individual feedback and its learning value was perceived higher. According to the students, the conditions for effective feedback were more often met in group feedback: the recognition of observable competencies (except physical examination), the use of standards and the provision of a plan of action to improve performance. Furthermore, individual feedback was more often used to correct performance deficiencies.

Several findings in this study can be related to the context of a collectivistic culture, for instance, that group feedback was appreciated more, that it was provided more often than individual feedback was, and especially more often by specialists [7, 11, 15]. That group feedback is provided and appreciated more than individual feedback may be explained from the fact that – in collectivistic cultures – group feedback serves to maintain harmony and integration in the group [11, 15]. The fact that students received group feedback more often from specialists may reflect the large power distance in Indonesia – a cultural dimension that refers to the degree of human inequality that underlies the functioning of a particular society [11] and that has been acknowledged to influence feedback processes [9, 16]. In cultures with a large power distance, hierarchy strongly influences relationship patterns, since superiors tend to maintain distance between themselves and their subordinates [11, 15]. In the clinical setting, specialists are higher in hierarchy than residents, and residents, in turn, are higher than students. Providing group feedback rather than individual feedback helps specialists to preserve the hierarchical distance. This power distance line of reasoning may also explain why students received individual feedback more often from residents than from specialists. Residents are closer to the students in the hierarchy than specialists are, because residents are also trainees in the clinical setting [10] and, hence, more eligible to seek individual feedback from residents than specialists [9].

Residents – compared to specialists – less often focused their feedback on clinical judgment. This may be explained from the fact that residents have less experience and, therefore, a lower level of competency than specialists have [17, 18], as a result of which they may feel less confident to focus their feedback on clinical judgment.

Group feedback focused more on all clinical competencies except on physical examination. This may be explained from the fact that feedback on physical examination requires the teacher to observe the individual student in action during the examination [19].

We found that in group feedback, specialists provided more often feedback on observable competencies than residents did. This might be because of the fact that specialists are more skilful and experienced in teaching.

We found that feedback on patient counselling, and on professional behaviour was mainly provided by specialists during group feedback. The explanation for this finding may be plural: first, patient counselling, and professional behaviour are complex competencies which have already been mastered by specialists, whereas residents may not yet be such professionals that they have enough confidence to give feedback on these aspects [17, 18]. Second, the group feedback setting may be considered as more suitable than individual feedback for teaching these competencies, since in a collectivistic culture, the most important motivation is meeting group expectations [20]. Giving this feedback in the group setting may emphasize the importance of acquiring these competencies and increase students’ motivation to improve their performance. Third, in a collectivistic culture, meeting the expectations of specialists as their teachers in clinical setting is an important motivating factor in student learning. The combination of receiving feedback from the specialist in the group setting may create a higher awareness of the significance of these competencies and stimulate the students to achieve higher competency levels.

In group feedback, we found that specialists compared students’ performance more often to the standards than residents did. This finding may be explained in a similar way as our findings with regard to patient counselling, and professional behaviour: higher competency levels of specialists [17, 18], the advantages of using the group setting to provide this kind of feedback and the influence of the specialists’ higher rank in the hierarchy as a motivating factor.

We found that – compared to individual feedback – group feedback less often included information to correct performance deficiencies. A possible explanation for this finding is that the individual feedback setting is more appropriate for correcting performance deficiencies since it offers more opportunities to be specific. The group setting is less suitable for correcting performance deficiencies, because group feedback requires more subtle and indirect ways of communication in order to avoid that students lose face because of making a mistake in front of other students, and, thus, to maintain harmony in the group [10, 11, 15]. Therefore, in group feedback, direct communication and discussing the performance of identified students in front of others should be avoided [11, 20].

Our study showed that group feedback more often contained a plan of action than individual feedback did. The objective of making a plan of action is that students apply the feedback in practice in order to narrow the gap between actual and desired performance [21, 22]. Again, the group setting may be more effective for providing a plan of action to improve performance, not only since it will be valued more [7, 11], but also because it may be interpreted as goal or target that has to be achieved by the group: in a collectivistic culture, people will control and regulate their personal goals to meet the goals of their groups [11, 20].

A limitation of our study was that it was based on students’ perceptions and not on actual learning outcomes. However, positive perceptions are a precondition to learning [23], which implies that student satisfaction with the learning value of feedback is vital to good learning outcomes. A second limitation is that Indonesia is a very large country in which cultural conditions can differ from region to region [24] so that our results may not be extrapolatable to other regions. However, the national group culture is reflected in the subcultures embedded in it [25] and, in general, cultural differences between countries are larger than those between subcultures within countries. So, our result may be generalizable to other medical schools in countries with collectivistic cultures. To ascertain the generalizability of the current outcomes, replication studies are needed.

The practical implication of our outcomes is that, in a collectivistic culture, group feedback adds to the array of educational measures that optimize students’ learning processes. Our findings may, therefore, add to the existent body of feedback literature, which mainly focuses on individual feedback in *individualistic* cultures, as it provides new insights from a different culture. It may be that congruence between culture and type of feedback is essential for the effectiveness of the feedback. We found that group feedback is highly valued by Indonesian students. However, individual feedback seems to focus distinctively on specific learning objectives. We are inclined to conclude that different kinds of feedback (individual versus group) seem to suit different purposes. Future research should focus on the importance of congruence between culture and educational concepts for the effectiveness of these concepts.

## Conclusion

We conclude that the concept of feedback as described in the literature, formulated from a rather individualistic perspective, is not comprehensive enough for the needs of collectivistic cultures. The degree to which group feedback satisfies the requirements for effective feedback and the high perceived learning value of group feedback underline the importance of group feedback.

## Abbreviations

CI: Confidence Interval
M: Mean
OR: Odds Ratio
SE: Standard error
